# Satisfaction with sex life and its impact on the quality of life in people living with HIV in Poland treated in the city of Bialystok: a cross-sectional study

**DOI:** 10.3389/fpsyt.2023.1270441

**Published:** 2023-09-13

**Authors:** Marta Milewska-Buzun, Mateusz Cybulski, Anna Baranowska, Elżbieta Krajewska-Kułak, Maria Kózka, Iwona Paradowska-Stankiewicz

**Affiliations:** ^1^Department of Integrated Medical Care, Faculty of Health Sciences, Medical University of Bialystok, Bialystok, Poland; ^2^Department of Clinical Nursing, Institute of Nursing and Midwifery, Faculty of Health Sciences, Jagiellonian University Medical College, Krakow, Poland; ^3^Department of Epidemiology of Infectious Diseases and Surveillance, National Institute of Public Health NIH – National Research Institute, Warsaw, Poland

**Keywords:** acceptance of illness, depression, human immunodeficiency virus (HIV), satisfaction with life, sexual life, quality of life

## Abstract

**Introduction:**

Sex life is an important element contributing to the overall quality of life. It is also a particularly sensitive dimension of quality of life for HIV-positive patients.

**Objective:**

The aim of the study was to assess the sexual life of people living with HIV in Poland treated in the Observation and Infection Clinic with the Subunit for HIV/AIDS Patients of the University Clinical Hospital in Bialystok, and its impact on the quality of life, life satisfaction, HIV status acceptance, general health status and depressive symptoms among the respondents.

**Methods:**

A total of 147 participants, including 104 men (70.7%) and 43 women (29.3%), took part in the research. The study was conducted between May 2019 and January 2020. The study used a diagnostic survey method with a modified questionnaire “Psychosocial situation of people living with HIV/AIDS” by Dr. Magdalena Ankiersztejn-Bartczak and the following standardised psychometric tools: the World Health Organization Quality of Life (WHOQOL-BREF), Short Form Health Survey (SF-36), Acceptance of Illness Scale (AIS), Satisfaction with Life Scale (SWLS), General Health Questionnaire (GHQ-28) and Beck Depression Inventory (BDI).

**Results:**

One-third of patients rated their sex life as poor or very poor. Almost half of respondents always informed sexual partners of their HIV status (49.7%). The sex life of respondents was highly correlated with almost all psychometric measures used in the study. Those indicating sexual contact as a possible source of HIV infection had a lower quality of life in the domain of general health compared to other respondents, but the difference was relatively small (about 5.5 points).

**Discussion:**

In conclusion, the overall satisfaction with the sex life of people living with HIV was moderate with a tendency to poor. The quality of life of people living with HIV was determined by their sex life. Better quality of life was presented by those with good self-reported sex life.

## 1. Introduction

HIV is one of the most prevalent health problems worldwide (1–3). It is estimated that 38 million people are HIV-positive globally (4). In Poland, 30,092 people had been diagnosed with HIV since the introduction of testing in 1985 until 31 December 2022 (5). Sex life is an important aspect of the quality of life (QoL). It is also a particularly sensitive QoL dimension for HIV-positive patients, with its determinants including clinical manifestations of HIV infection, adverse effects of antiretroviral therapy, social stigma associated with HIV status and the fear of transmission, despite the fact that those on effective, modern treatment no longer pose a threat in terms of viral transmission (6). Studies conducted so far have shown that the proportion of HIV patients reporting a satisfying sex life seems to be lower than in other social groups (7–10). In Poland, no representative studies on this issue have been conducted so far and the available publications are scarce.

Therefore, the aim of this study was to evaluate the sexual life of people living with HIV in Poland treated in the Observation and Infection Clinic with the Subunit for HIV/AIDS Patients at the Teaching Hospital of the Medical University in Bialystok, and its impact on the quality of life, satisfaction with life, HIV status acceptance, general health status and depressive symptoms among the respondents.

We hypothesized that satisfaction with sex life in the study group would be poor. In addition, we assumed that satisfaction with sex life would be an important factor determining the quality of life of HIV-positive people. To verify the hypotheses mentioned above, the following research questions were formulated:

1. What is the general level of satisfaction with sex life in the study group of patients?
2. Does satisfaction with sex life affect the quality of life in general and its particular domains (satisfaction with life, acceptance of infection, occurrence of depressive symptoms, general health status)?

## 2. Materials and methods

### 2.1. Study group

The analysis included people living with HIV treated in the Observation and Infection Clinic with the Subunit for HIV/AIDS Patients of the Department of Infectious Diseases and Hepatology of the Teaching Hospital of the Medical University in Bialystok and the Consultation and Diagnostic Centre at the Teaching Hospital of the Medical University of Bialystok. A total of 147 people, including 104 men (70.7%) and 43 women (29.3%), took part in the study. The mean age of the respondents ( $\bar{x}$ ) was 42.5 years with a standard deviation (SD) of 10.4 years. The youngest among the patients surveyed was 22 years old, while the oldest respondent was 77 years old. Respondents with secondary education (36.1%) were the predominant group. Urban residents, with a predominance of those living in cities with more than 200,000 inhabitants (residents of Bialystok), dominated in the study group. Rural respondents accounted for less than 14%. Almost one in two patients were single. Married respondents accounted for almost 30% of the surveyed group. People who were not currently in any relationship (40.1%) predominated in the study group. Non-single patients tended to report an informal relationship. The majority of respondents had children, with the largest group reporting one or two children. However, a large proportion of patients did not have children (48%). Those who were economically active accounted for more than half of the surveyed group (55.8%). The majority of respondents described their financial status as moderate (34.7%) or good (40.8%). The mean duration of infection was almost 12 years (11.8 ± 7.7), with less than 10 years since infection in half of the study group. Detailed data are shown in Table 1.

**Table 1:** Sociodemographic characteristics of respondents.

Sociometric variable		*n*	%
Gender	Female	43	29.3%
	Male	104	70.7%
Age (years)	<30	14	9.5%
	30–39	50	34.0%
	40–49	51	34.7%
	50–59	22	15.0%
	60–69	8	5.4%
	≥70	2	1.4%
Education	Primary	31	21.1%
	Vocational	37	25.2%
	Secondary	53	36.1%
	Higher	26	17.7%
Place of residence	Rural	20	13.6%
	City up to 50,000	29	19.7%
	City of 50–100,000	39	26.5%
	City of 100–200,000	13	8.8%
	City >200,000	46	31.3%
Marital status	Single	71	48.3%
	Married	42	28.6%
	Divorced	28	19.0%
	Widow/widower	6	4.1%
Type of current relationship	Formal	40	27.2%
	Informal	48	32.7%
	No relationship	59	40.1%
Number of children	One	35	23.8%
	Two	23	15.6%
	Three	7	4.8%
	Four and more	12	8.2%
	No children	70	47.6%
Professional activity^1^	Employed	82	55.8%
	Disability pensioner	29	19.7%
	Student	15	10.2%
	Retired	9	6.1%
	Jobseeker	9	6.1%
	Non-jobseeker	8	5.4%
	Not working for health reasons	8	5.4%
Material situation	Very bad	1	0.7%
	Bad	25	17.0%
	Neither good nor bad	51	34.7%
	Goods	60	40.8%
	Very good	10	6.8%

^1^ The sum does not have to equal 100% as any number of response options could be indicated.

### 2.2. Study design

The study was conducted between May 2019 and January 2020 in the Observation and Infection Clinic with the Subunit for HIV/AIDS Patients and the Consultation and Diagnostic Centre of the Teaching Hospital of the Medical University of Bialystok. The inclusion criteria were as follows confirmed HIV infection, a stay in a hospital ward or a visit to the Consultation and Diagnostic Centre, and an informed and voluntary consent to participate in the study. The study was approved by the Management of the Institution and the Head of the Department. Patients’ rights, including the right to intimacy and anonymity, were respected. In order to meet all ethical requirements during the implementation of the study, each respondent made a voluntary decision to take part in the study and could also withdraw from the study at any stage. The respondents completed the questionnaire unassisted due to the very personal nature of the questions, mainly contained in the survey; however, they were informed that if any doubts or problems with understanding the questions should arise, they could ask for clarification. Each patient hospitalised or presenting at the Diagnostic and Consultation Centre was asked to complete the questionnaires (in paper form) by themselves. Additionally, it was explained that the data obtained would only be used for research purposes. Patients completed the questionnaires in the patient room (in-patients), at the Consultation and Diagnostic Centre, or at home and handed them in during the next visit. The study was conducted with the involvement of persons in close contact with the HIV-positive patients, i.e., infectious disease doctors, nurses, and during direct meetings with HIV patients. A total of 198 questionnaires were distributed, of which 159 (80.30%) were returned, including 12 incomplete questionnaires (19.08%), which were discarded during the analysis. A total of 147 questionnaires were included in the analysis – survey return rate of 74.25%.

### 2.3. Measures

The study used the method of a diagnostic survey with the use of a modified questionnaire “Psychosocial situation of people living with HIV/AIDS in Poland” by Dr. Magdalena Ankiersztejn-Bartczak, President of the Social Education Foundation in Warsaw. Written consent of the author was obtained for the use of the questionnaire. Additionally, the following standardised psychometric tools were used in the study: The World Health Organization Quality of Life (WHOQOL-BREF), Short Form Health Survey (SF-36), Acceptance of Illness Scale (AIS), Satisfaction with Life Scale (SWLS), General Health Questionnaire (GHQ-28) and Beck Depression Inventory (BDI).

#### 2.3.1. Modified questionnaire of the survey “psychosocial situation of people living with HIV/AIDS in Poland”

The survey questionnaire consisted of 59 questions (11). All questions required a specific choice of one or more answers. Some questions were additionally open-ended, giving the respondent the opportunity to address the question more broadly or to voice his/her own view/suggestion. The questions were structured in a way that was clear and comprehensible for the patient, and referred to a retrospective analysis of the situation since receiving the diagnosis and an assessment of various aspects of life, including those relating to the last twelve months only. The questions in the questionnaire were grouped into four thematic categories:

1. Socio-demographic characteristics, including age, education, place of residence, income, housing conditions.
2. Diagnosis and confirmation of HIV infection.
3. The impact of the diagnosis on life.
4. Public reactions to information about infection.

#### 2.3.2. The world health organization quality of life (WHOQOL-BREF)

The WHOQOL-BREF questionnaire contains 26 questions and is used to measure quality of life in four domains: psychological health, physical health, environment and social relationships (12). The psychological domain includes positive and negative feelings, physical appearance, religion and spirituality, self-esteem, faith, sense of concentration, thinking, memory and learning. The physical domain includes rest and sleep, discomfort and pain, mobility, daily activities, dependence on medication and treatment, and ability to undertake work (12). In the environmental domain, respondents assessed their financial resources, sense of security, freedom, access to and quality of health care, relationships with the immediate environment, housing conditions, opportunities for rest and recreation, opportunities for acquiring new information and skills, and transport. The social domain includes interpersonal relationships, satisfaction with sexual life, and social support (12). Additionally, the WHOQOL-BREF contains two questions that are analysed separately. Question 1 asks about an individual’s overall perception of quality of life, and question 2 asks about an individual’s overall perception of their health. Responses are scored on a 5-point scale (low score of 1 to high score of 5), with a reverse interpretation in three questions, i.e., 5 is the lowest value and 1 is the highest value. A maximum score of 20 can be obtained in each of the domains indicated above. The higher the score, the better the patients’ quality of life (12). Cronbach’s alpha coefficient values for each of the six domains range from 0.71 (for the social domain) to 0.86 (for the environmental domain). The overall Cronbach’s alpha coefficient for the scale is 0.84 (12).

#### 2.3.3. Short form health survey (SF-36)

The SF-36 Quality of Life Assessment Questionnaire was created in 1988 and is one of the most widely used generic tools for measuring health-related quality of life. It is designed for subjective assessment of health status (13). Due to its high diagnostic sensitivity, it can be used even in the early stages of disease. The tool consists of 36 questions in 11 categories to distinguish eight aspects of quality of life, such as:

- physical function – range of typical physical daily activities (10 items);
- role limitations due to physical problems – the effect of physical health on daily activities (4 items);
- bodily pain – severity of physical pain and its impact on daily activities (2 items);
- general health perceptions – i.e. the patient’s self-reported overall health in relation to their expectations and perception of health (5 items);
- vitality – level of vital energy and fatigue (4 items);
- social functioning – impact of health on social functioning (2 items);
- role limitations due to emotional problems – impact of emotional problems on daily functioning (3 items);
- perceived mental health – quantitatively classified as nervousness, irritability, depression, happiness (5 items) (13).

Additionally, health status is assessed in comparison with the health status one year before. The type of answers to individual questions varies from dichotomous (yes/no) to 3-, 5- and 6-point Likert scales. Respondents’ answers are normalised so that the resulting QoL measures range from 0–100, with 0 always indicating the worst QoL and a score of 100 indicating the best QoL. Cronbach’s alpha coefficient values range from 0.73 (social functioning) to 0.96 (role limitations due to physical health, role limitations due to emotional problems and vitality) (13).

#### 2.3.4. Acceptance of illness scale

The AIS questions address specific difficulties and limitations arising from one’s health status. The AIS can be used to measure acceptance of any illness. It contains eight statements describing negative health consequences in the form of limitations due to the illness, lack of self-sufficiency, the sense of being dependent on others and reduced self-esteem (14). In each statement, the respondents identify their current health status on a five-point Likert scale, where: 1 – strongly agree, 2 – agree, 3 – not sure, 4 – disagree, and 5 – strongly disagree. Strongly agree indicates poor adaptation to the disease, while disagree indicates disease acceptance. The overall score ranges from 8 to 40. The degree of acceptance is defined by three score ranges. A score of 8 to 18 indicates a lack of illness acceptance, 19 to 29 represents an average level of acceptance, and 30 to 40 defines a high level of acceptance of the health situation. The reliability of the Polish version of the AIS is similar to that of the original version, with a Cronbach’s alpha coefficient of 0.82 (14).

#### 2.3.5. Satisfaction with life scale

The Satisfaction with Life Scale (SWLS) consists of five statements, which are rated by the respondent on a 7-point scale by selecting one of the possible answers. The respondent assesses to what extent each of the statements applies to his or her life to date, where: 1 – strongly disagree, 2 – disagree, 3 – slightly disagree, 4 – neither agree nor disagree, 5 – slightly agree, 6 – agree, and 7 – strongly agree (15). The answers are scored and the total score represents the overall degree of satisfaction with life. The scores range from 5 to 35, and the higher the score, the greater the sense of satisfaction with life. Sten scale is used for interpretation, where scores between 1–4 stens (a score of 5–17) represent low values, 5–6 stens (a score of 18–23) represent average values, and 7–10 stens (a score of 24–35) represent high values. A score of 20 represents a neutral point on the scale and means that the respondent is neither satisfied nor dissatisfied to any degree. A score of 5–9 indicates extreme dissatisfaction with life, while a score of more than 30 indicates high satisfaction with life. The Cronbach’s alpha coefficient is 0.87 (15).

#### 2.3.6. General health questionnaire (GHQ-28)

The General Health Questionnaire was created by D. Goldberg as a screening tool to identify individuals at risk of non-psychotic mental disorders, as well as assess their severity (16). The GHQ-28 questionnaire has four 7-item sub-scales: somatic symptoms, items 1–7 (GHQ-28-A); anxiety/insomnia, items 8–14 (GHQ-28-B); social impairment, items 15–21 (GHQ-28-C); and depressive symptoms, items 22–28 (GHQ-28-D). The questionnaire is one of the so-called self-report tools, in which the respondent answers the questions independently by choosing one of the given options (i.e., better than usual; same as usual; worse than usual; much worse than usual). Each item is scored from 0 to 3. The maximum score is 84. The higher the total score, the higher the risk of a non-psychotic mental disorder, with the threshold for suspicion at 23/24 (16). The Cronbach’s alpha coefficient for the scale oscillates between 0.9 and 0.95 (17).

#### 2.3.7. Beck depression inventory

The BDI was developed by Beck et al. (18). It is a self-report tool for assessing the severity of depressive symptoms. It is widely used not only in psychiatric disorders, but also in internal medicine, oncology, urology, gynaecology or neurology to assess patients’ mood (18). The BDI is used as a screening diagnostic tool to measure the severity of depressive symptoms, monitor its dynamics, as well as assess the efficacy of pharmacotherapy and psychotherapy. The scale consists of 21 sets of statements scored from 0 to 3 (severity). For each item, the respondent chooses one answer that, in his or her opinion, best describes his or her situation in a given time period. The total summed score can range from 0 to 63, with higher values indicating greater severity of depression. Scores are also classified into four ranges. Depending on the score obtained, the severity of depression can be determined: 0–11 no depression; 12–26 mild depression; 27–49 moderate depression; 50–63 severe depression. The Cronbach’s alpha coefficient is 0.86 (18).

### 2.4. Procedure and ethical considerations

The study was carried out following the recommendations and was reviewed and approved by the Bioethics Committee of the Medical University in Bialystok (statute no. R-I-002/237/2019). All subjects gave written informed consent in accordance with the Declaration of Helsinki.

### 2.5. Statistical analysis

Descriptive statistics and statistical inference, with the choice of methods determined by the type and distribution of the characteristics analysed, were used for statistical analysis.

The descriptive section presents the numerical and percentage distribution of nominal characteristics, while for measurable characteristics (mainly psychometric measures) selected descriptive statistics were determined: arithmetic mean ( $\bar{x}$ ), median (middle value) (Me), maximum value (max.) and minimum value (min.), standard deviation (SD) and lower and upper quartile (*c*_25_ and *c*_75_).

When psychometric measures were compared between groups, i.e., when the independent factor was nominal, descriptive statistics were determined in the compared groups and the significance of the differences between them was assessed using the Mann–Whitney test for two groups.

The results of all the above-mentioned statistical tests were interpreted using the probability (*p*) value, assuming a statistically significant relationship at *p* < 0.05.

## 3. Results

Table 2 shows the respondents’ level of satisfaction with their sex life. About 34% of patients rated their sex life as poor or very poor. The same percentage of respondents described their sex life as average, while 31% of respondents were satisfied with their sex life.

**Table 2:** The level of sex life satisfaction.

Sex life	*n*	%
Very poor	35	23.8%
Poor	15	10.2%
Neither good nor poor	50	34.0%
Good	21	14.3%
Very good	25	17.0%
No answer	1	0.7%

Almost half of respondents always informed their sexual partners of their HIV status (49.7%). This was never done by 13% of those infected. Almost half of respondents (46.3%) always used a condom as a form of protection against HIV. The detailed distribution is shown in Table 3.

**Table 3:** Informing sexual partners about HIV status and condom use by respondents.

	*n*	%
Informing sexual partners about HIV status		
Never	19	12.9%
No, if I use a condom	16	10.9%
Not always	17	11.6%
Always	73	49.7%
No answer	22	15.0%
Use of condoms		
Always	68	46.3%
From time to time	26	17.7%
Rarely	11	7.5%
Never	20	13.6%
No answer	22	15.0%

The question in Table 4 addressed only patients who did not inform their sexual partners about their HIV-positive status. Up to 65.3% of respondents could not account for such behaviour. The others pointed to fear of discrimination, such as rejection by the partner, spreading information about the infection, shame, loneliness and hostility. The use of techniques that do not pose a risk of transmission was reported by 16.3% of respondents, while 4.8% of participants felt that this was solely their concern.

**Table 4:** Reasons for not informing sexual partners about HIV status.

Reasons for not informing partners about HIV status	*n*	%^1^
Use of safe techniques	24	16.3%
Not know how to say this	23	15.6%
Fear of rejection	23	15.6%
Fear of spreading information about the infection	20	13.6%
Shame	16	10.9%
Fear of mistreatment	13	8.8%
Fear of being alone	13	8.8%
Fear of being rejected	8	5.4%
Fear of hostility	8	5.4%
This is only my concern	7	4.8%
None of the above	96	65.3%

^1^ The sum does not have to equal 100% as any number of response options could be indicated.

It was shown that the self-reported sex life was highly correlated with almost all the psychometric measures considered in this paper. The higher rated the sex life, the higher the quality of life, HIV status acceptance, satisfaction with life and general health status, and the lower the level of depressive symptoms (Table 5).

**Table 5:** Self-reported sex life and QoL by SF-36, WHOQOL-BREF, GHQ-28, AIS, SWLS, and BDI.

Psychometric measures		Self-reported sex life									*p*
		poor (*n* = 71)			average (*n* = 42)			good (*n* = 34)			
		$\bar{x}$	Me	SD	$\bar{x}$	Me	SD	$\bar{x}$	Me	SD	
SF-36	Physical function	62.2	62.5	32.6	85.9	95.0	22.7	90.1	95.0	14.4	0.0000*
	Role limitations due to physical health problems	31.5	0.0	42.2	74.5	100.0	39.3	73.9	100.0	33.3	0.0000*
	Pain	50.9	45.0	36.6	75.0	90.0	25.1	70.6	78.8	22.2	0.0031*
	General health	42.5	50.0	25.5	47.0	50.0	19.5	50.8	50.0	13.3	0.3420
	Role limitations due to emotional problems	50.0	50.0	47.7	73.3	100.0	41.0	88.4	100.0	29.2	0.0001*
	Vitality	35.5	31.3	14.9	48.9	50.0	12.0	43.9	50.0	12.5	0.0000*
	Social functions	61.5	62.5	26.6	79.7	87.5	22.6	81.8	93.8	21.8	0.0001*
	Wellbeing	40.9	42.2	21.6	58.6	56.7	17.2	65.5	63.3	16.4	0.0000*
	Physical area	51.9	51.7	28.4	77.8	88.5	23.0	80.0	82.8	14.1	0.0000*
	Mental domain	43.7	43.5	17.9	61.2	63.9	14.5	66.3	69.3	14.1	0.0000*
	Total quality of life	47.8	50.6	21.5	69.5	76.6	16.8	73.1	74.7	12.8	0.0000*
WHOQOL-BREF	Somatic domain	12.3	12.9	3.2	15.6	16.0	2.5	15.5	16.0	2.2	0.0000*
	Psychological domain	10.2	10.0	3.3	13.3	13.3	2.7	14.1	14.7	2.4	0.0000*
	Social domain	9.6	9.3	3.3	13.3	14.7	3.0	15.5	16.0	3.2	0.0000*
	Environment	13.1	13.5	2.8	14.2	14.8	2.2	15.7	16.0	1.6	0.0000*
GHQ-28	Somatic symptoms	3.2	2.5	2.6	1.4	1	1.9	1.3	0	2.0	0.0000*
	Anxiety, insomnia	3.1	4	2.7	1.3	0	2.0	1.4	0	2.0	0.0007*
	Social dysfunctions	2.5	2	2.5	0.6	0	1.4	0.9	0	1.7	0.0000*
	Depressive symptoms	1.5	1	1.9	0.6	0	1.4	0.6	0	1.6	0.0020*
	Total	10.2	10	8.3	3.9	1	5.7	4.2	2	6.0	0.0000*
SWLS		13.5	12	7.6	19.7	21	7.9	20.5	22	7.0	0.0000*
AIS		24.6	24.5	9.7	28.4	30	9.3	31.6	35	8.1	0.0016*
BDI		20.8	17	14.6	7.3	4.5	8.8	5.7	3.5	6.5	0.0000*

AIS – acceptance of illness scale, BDI – beck depression scale, GHQ-28 – general health questionnaire, SF-36 – quality of life assessment questionnaire, SWLS – satisfaction with life scale, WHOQOL-BREF – world health organisation abbreviated quality of life questionnaire, $\bar{x}$ – arithmetic mean, Me – median, SD – standard deviation, *p* – probability value, * – statistically significant.

The vast majority of respondents (*n* = 92, 62.6%) contracted HIV through sexual contact. Therefore, we investigated whether those reporting sexual route of transmission presented with a different quality of life than the others. It was shown that the route of infection had essentially no effect on current QoL. Those reporting sexual contact as a possible source of HIV infection had lower QoL in the general health domain than the other respondents, but the difference was only minor (about 5.5 points). This was the only statistically significant relationship for the SF-36 measures. For the WHOQOL-BREF measures, there was one difference that was close to statistically significant – those indicating possible infection through sexual contact had a slightly lower QoL in the psychological domain (Table 6).

**Table 6:** Sexual route of HIV transmission and QoL by SF-36 and WHOQOL-BREF.

Psychometric measures		Sexual contacts						*p*
		yes (*n* = 92)			no (*n* = 55)			
		$\bar{x}$	Me	SD	$\bar{x}$	Me	SD	
SF-36	Physical function	78.2	95.0	27.0	81.1	95.0	28.1	0.2709
	Role limitations due to physical health problems	58.4	75.0	42.5	62.3	100.0	45.1	0.3438
	Pain	63.9	77.5	30.4	68.3	77.5	30.7	0.3788
	General health	44.8	50.0	19.3	50.2	50.0	22.0	0.0405*
	Role limitations due to emotional problems	71.0	100.0	42.0	69.1	100.0	44.8	0.9925
	Vitality	43.4	50.0	14.7	41.8	43.8	13.7	0.5359
	Social functions	72.0	75.0	25.5	78.0	87.5	24.8	0.1693
	Wellbeing	53.9	51.1	21.8	55.9	57.8	20.2	0.3444
	Physical area	68.5	76.7	25.5	72.1	78.6	27.0	0.2648
	Mental domain	56.5	61.1	19.0	57.4	60.0	17.1	0.8570
	Total quality of life	62.5	69.0	21.0	64.7	71.1	20.2	0.6698
WHOQOL-BREF	Somatic domain	14.4	14.6	3.2	14.6	15.4	3.0	0.3576
	Psychological domain	12.2	12.7	3.4	13.1	13.3	3.0	0.0922
	Social domain	12.8	13.3	4.0	12.5	13.3	4.0	0.6315
	Environment	14.2	14.5	2.4	14.4	15.0	2.6	0.3499

SF-36 – quality of life assessment questionnaire, WHOQOL-BREF – world health organisation abbreviated quality of life questionnaire, $\bar{x}$ – arithmetic mean, Me – median, SD – standard deviation, *p* – probability value, * – statistically significant.

## 4. Discussion

A healthy and satisfying sex life is considered an important factor determining good health and a satisfying quality of life. Substance and alcohol abuse, low self-esteem, shame and fear of HIV transmission can negatively affect the sexual function of HIV-positive individuals (19). Studies indicate that sexual problems and dysfunctions are more common in people with HIV than in the healthy population, regardless of gender (20). Therefore, people living with HIV continue to face problems with intimacy and physical pleasure. Typically, sexual dysfunctions and specific aspects of sexuality related to HIV remain of secondary importance in daily clinical practice compared to the treatment of HIV infection itself (21). Ignoring sexual problems compromises the quality of life of these patients, which may already be significantly reduced by HIV-related stress, associated diseases and stigma (22). In men, sexual dysfunctions, erectile problems in particular, are more common in people living with HIV than in healthy individuals. Since these dysfunctions further reduce QoL and lead to poor adherence (23), it seems important to include sexology professionals in the HIV treatment team (23). Almost half of the sexually active respondents (49.7%) always informed their sexual partners about their HIV status; 12.9% of respondents never shared this information, while 11.6% not always informed their sexual partner about their HIV infection. The use of safe sexual techniques that did not carry the risk of viral transmission (16.3%) was the most commonly reported reason, while 15.6% of respondents said they did not know how to tell their partner and did not do so for fear of rejection. This was followed by fear of the information about their HIV status being spread, shame, fear of being mistreated, hostility and loneliness. Seven people felt that it was solely their own concern. A total of 50 respondents rated their sex life as good or very good, another 50 rated it as average, while 46 respondents described their sex life as poor, bad or very bad. One person did not answer the question. The relationship between sex life and the QoL of respondents was also assessed. It was found that sex life was highly correlated with almost all psychometric measures considered in the study. The higher the self-reported sex life satisfaction, the higher the quality of life scores, the lower the severity of depressive symptoms, the higher the acceptance of HIV status and satisfaction with life, as well as the lower the level of depressive symptoms. In their study in 692 people living with HIV in France, Peyre et al. (24) showed that the respondents’ quality of sexual life was poor and largely determined by concerns about the potential transmission of the virus to a partner. Poorer quality of sex life was significantly associated with female gender, lack of a stable sexual partner, unemployment, low income, perceived fear of HIV transmission, lower self-esteem and overall poor health (24). Factors determining the quality of life of people living with HIV were also analysed by Osei-Yeboah et al. (25). The study group consisted of 158 patients on cART. The WHOQOL-HIV-BREF questionnaire was used for QoL assessment. The authors found that sexual inactivity, especially among men, was associated with poorer health-related quality of life (25). Shacham et al. conducted a study to assess the sexual life of HIV-positive people and identify social factors determining its quality (20). The study used, among others, the Sexual Function Questionnaire (SFQ). They found that sexual function was significantly lower in the group of HIV patients than in the group of cancer survivors in all subscales, except for masturbation. Both women and men presented with lower quality of sex life, which significantly correlated with depressive symptoms, indicating a lower quality of life (20).

In summary, research has shown that HIV-positive individuals who experience sexual dysfunctions or poor sexual satisfaction often also have a lower quality of life, higher levels of depressive symptoms and greater difficulty managing their disease. This can lead to non-adherence to medical treatment, including antiretroviral treatment, which in turn increases the risk of HIV transmission and exacerbates the symptoms of infection. On the other hand, HIV-positive individuals with good quality of sex life are likely to present with better perceived general health, greater motivation to comply with medical advice and better overall health; therefore a comprehensive approach to sexuality in this patient group should be considered in HIV/AIDS health programmes.

### 4.1. Limitations

Research on the quality of life in HIV-positive persons is a complex process that requires specialised knowledge, interpersonal skills and, above all, the maintenance of a high ethical level at every stage of the study. In order to make the above assessment as reliable as possible, it becomes necessary to study many aspects of life, including the intimate sphere, which is often an extremely difficult subject for HIV-positive patients. The assistance in completing the survey and questionnaires offered to patients was not met with respondents’ approval. It should be emphasised that in order to maintain a high ethical standard, respondents were informed that they could withdraw from the study at any stage.

Analysing the research data, it was found that one or more questions, mainly related to the intimate sphere, were not answered. Given the very broad scope of the study, and therefore the necessity to complete an extensive survey and four questionnaires (SF-36, AIS, WHOQOL-BREF, SWLS), it can be assumed that some respondents may have consciously skipped selected questions. It can also be assumed that this may have resulted from accidental or deliberate omissions, due to the overly intimate nature of the questions or the voluminous nature of the questionnaire. The questionnaires completed by the respondents were handed over to the medical staff (doctor, nurse) involved in the patient’s therapy. Although each questionnaire was prepared in such a way that those receiving it from the patients did not have direct access to the contents (a sealed return envelope was used), it can be assumed that some respondents may have had concerns about whether the confidentiality principle applicable to the survey would actually be maintained. In the future, consideration should be given to providing a specially prepared and secured box for this purpose. It is also possible that some questions were incomprehensible to respondents. The place where the survey was completed may also have been the reason why some questions were left unanswered. Some respondents took the questionnaire home, while others did not for personal reasons. This group chose to complete the questionnaire either while at the doctor’s appointment (in a specially prepared room) or in the patient room (the group being hospitalised). This could have been a reason for respondents to be distracted or to fill in the forms superficially, without deeper reflection, due to haste or impatience (having to return to work, home duties, or due to public transport schedules). Also, some of those who decided to take the questionnaire home did not return them. It must be assumed that they simply did not intend to take part in the survey. Therefore, the quality of life of patients treated in the Observation and Infection Clinic with the Subunit for HIV/AIDS Patients at the Teaching Hospital of the Medical University of Bialystok does not refer to the total group of seropositive individuals attending the Centre. In the future, it would be advisable to consider involving interviewers who would provide assistance in completing the questionnaires or using fewer survey tools.

In conclusion, the overall satisfaction with the sex life of people living with HIV was moderate with a tendency to poor. The quality of life of HIV-positive patients was determined by the level of their sex life. A higher quality of life was presented by people with better self-reported sex life. Moreover, the higher rated the sex life, the higher HIV status acceptance, satisfaction with life and general health status, and the lower the level of depressive symptoms. Low level of HIV status acceptance was observed among those with poor self-reported quality of sex life.

## Data availability statement

The raw data supporting the conclusions of this article will be made available by the authors, without undue reservation.

## Ethics statement

The studies involving humans were approved by the Bioethics Committee of the Medical University in Bialystok. The studies were conducted in accordance with the local legislation and institutional requirements. The participants provided their written informed consent to participate in this study.

## Author contributions

MM-B: Conceptualization, Data curation, Formal analysis, Investigation, Methodology, Project administration, Writing – original draft. MC: Conceptualization, Funding acquisition, Methodology, Writing – original draft, Writing – review & editing. AB: Data curation, Investigation, Writing – original draft. EK-K: Writing – review & editing. MK: Writing – review & editing. IP-S: Writing – review & editing.

## Funding

The author(s) declare that no financial support was received for the research, authorship, and/or publication of this article.

## Conflict of interest

The authors declare that the research was conducted in the absence of any commercial or financial relationships that could be construed as a potential conflict of interest.

## Publisher’s note

All claims expressed in this article are solely those of the authors and do not necessarily represent those of their affiliated organizations, or those of the publisher, the editors and the reviewers. Any product that may be evaluated in this article, or claim that may be made by its manufacturer, is not guaranteed or endorsed by the publisher.
